# Internet-based profiler system as integrative framework to support translational research

**DOI:** 10.1186/1471-2105-6-304

**Published:** 2005-12-19

**Authors:** Robert Kim, Francesca Demichelis, Jeffery Tang, Alberto Riva, Ronglai Shen, Doug F Gibbs, Vasudeva Mahavishno, Arul M Chinnaiyan, Mark A Rubin

**Affiliations:** 1 Department of Pathology, Brigham and Women's Hospital, Boston, USA; 2 Harvard Medical School, Boston, USA; 3 Children's Hospital Informatics Program, Children's Hospital, Boston, USA; 4 Department of Biostatistics, University of Michigan School of Public Health, Ann Arbor, USA; 5 Department of Pathology, University of Michigan, Ann Arbor, USA; 6 Department of Urology, University of Michigan, Ann Arbor, USA; 7 Dana Farber Harvard Comprehensive Cancer Center, Boston, USA

## Abstract

**Background:**

Translational research requires taking basic science observations and developing them into clinically useful tests and therapeutics. We have developed a process to develop molecular biomarkers for diagnosis and prognosis by integrating tissue microarray (TMA) technology and an internet-database tool, Profiler. TMA technology allows investigators to study hundreds of patient samples on a single glass slide resulting in the conservation of tissue and the reduction in inter-experimental variability. The Profiler system allows investigator to reliably track, store, and evaluate TMA experiments. Here within we describe the process that has evolved through an empirical basis over the past 5 years at two academic institutions.

**Results:**

The generic design of this system makes it compatible with multiple organ system (e.g., prostate, breast, lung, renal, and hematopoietic system,). Studies and folders are restricted to authorized users as required. Over the past 5 years, investigators at 2 academic institutions have scanned 656 TMA experiments and collected 63,311 digital images of these tissue samples. 68 pathologists from 12 major user groups have accessed the system. Two groups directly link clinical data from over 500 patients for immediate access and the remaining groups choose to maintain clinical and pathology data on separate systems. Profiler currently has 170 K data points such as staining intensity, tumor grade, and nuclear size. Due to the relational database structure, analysis can be easily performed on single or multiple TMA experimental results. The TMA module of Profiler can maintain images acquired from multiple systems.

**Conclusion:**

We have developed a robust process to develop molecular biomarkers using TMA technology and an internet-based database system to track all steps of this process. This system is extendable to other types of molecular data as separate modules and is freely available to academic institutions for licensing.

## Background

Taking laboratory discoveries and translating them into clinically useful diagnostic tests or targeted therapies requires the use of human samples for validation. This process, referred to as translational research, requires carefully storing and maintaining detailed annotation of these samples. Tissue Microarray (TMA) technology has created and efficient manner to accelerate discovery but has also created a new demand for databases to handle a large number of data points in a regulatory compliant manner.

The initial description of Tissue Microarray (TMA) technology by Kononen et al. [1] began a revolutionary change in the way that many tissue based research studies would be performed. TMA technology allows for the precise placement of typically up to 400–800 tissue samples into a single block in an array. The approach some major advantages as compared to using standard slides. TMA technology decreases experimental variability, conserves tissue resources and provide a dramatic cost benefit once the initial TMA block is constructed [2].

TMA technology also presents some challenges, which include issues related to sampling strategies [3-5], evaluation of TMA samples[6,7], ability to store and maintain the data derived from TMA experiments [8-11], and analysis of TMA datasets [12]. All of these issues are important to understanding the utility of TMA technology and to help maximize its potential as a research tool. In the collaborative setting of the Specialized Program of Research Excellence (S.P.O.R.E.) for Prostate Cancer, a program for translational research, pathologists from the University of Michigan, Baylor School of Medicine, and Johns Hopkins developed a working model to deal with these challenges. We recognized some fundamental guiding principles: (1) TMA technology provides a means to conserve finite tissue resources, while at the same time increasing the number of studies for qualified investigators; (2) TMA technology is most valuable if data generated from each experiment is recorded and returned to a central database thus increasing the annotation and value of each sample; and (3) TMA technology is ideally suited for collaborative biomarker research. These important guidelines suggested to our working group that TMA databasing tools would be a cornerstone to our translational research activities and would grow in importance over time and with the increasing number of experimental data.

**Figure 1:**
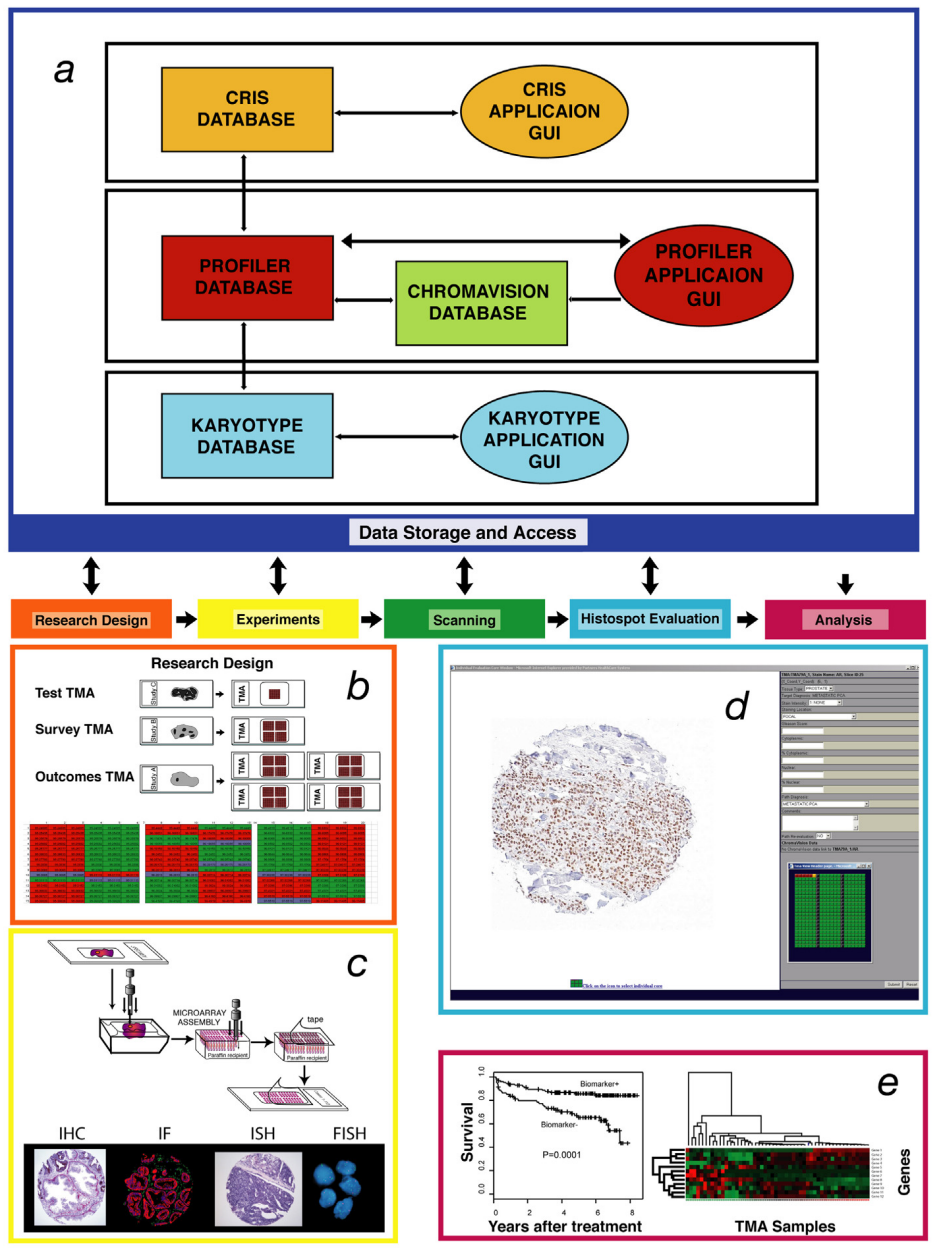
a-e. Profiler System Organization and Workflow for TMA Module. Component view of the architecture of the Profiler framework(a). The Profiler interfaces with multiple components besides TMA data. For example, the Clinical Research Information System (CRIS) component manages clinical data on patients being taking care of at the Dana Farber Cancer Institute and the Brigham and Women's Hospital. Using regulatory compliant protocols, appropriate consented cases information can be linked to the system. The karyotype component manages cytogenetic data. Different components are linked by means of unique identifiers (e.g., research coded identifier). The current system contains modules for karyotype, expression array, SNP array (not shown), and tissue microarray (TMA) data. Users can access different components using Graphical User Interfaces (GUI) at local workstations. The process of TMA use can for research can be divided into 5 steps, which include the following: research design, experiments, scanning of histospot images, histospot evaluation, and analysis. The profiler system is designed to store data for all 5 steps of this process. The first step in the work flow of a TMA study is the research design (b). As described in the text, each TMA study design is based on the questions being addressed. Three examples are given in this schematic view of research design. Test TMAs are usually small TMA with a small number (i.e., n = 100) cores. The test arrays are useful for working up reagents. These arrays provided limited information regarding protein expression or RNA expression and represent an efficient method of conserving tissues. Survey TMAs allow for determination of expression on a wide range of samples. Standard survey TMAs have approximately 500 histospots. An outcomes TMA may include a large number of samples with outcomes information. These usually represent the most valuable samples given the annotation of each case. As described in the results section, organization of the paraffin tissue blocks (upper right), circling of the areas of interest on standard slides and construction of the TMA are the final steps of developing a TMA for experiments. After the conceptual design of the TMA experiment, a physical map (TMA Map, bottom) is draw based on the available tissue blocks. The research identifiers from these blocks are linked to the research patient identifiers. Part of the experiment includes the construction of TMA (e, top). Glass slides and blocks are organized, the pathologist circles the areas of interest and tissue cores are taken from the donor block and placed into a tissue microarray. Typical types of experiments that can be performed using TMAs include immunohistochemistry (IHC), immunofluorescence (IF), in situ hybridization (ISH), and Fluorescence In Situ Hybridization (FISH) (c, bottom). Following experiments with IHC, histospot are scanned (not shown) and the data is evaluated using the Internet interface of Profiler (d). As described in the text, the system allow the reviewer to view the histospot images at several magnifications (shown is 10 ×, also available are 2.5 ×, 5 ×, and 20 × magnification). Data can be added by the reviewer (d, right side). The reviewer can also see the TMA map design (d, bottom). The growing annotated data on a TMA makes it more valuable over time. Analysis is performed on the data usually outside of the Profiler system using standard statistical analysis tools (e). Type of results that can be evaluated using TMA technology include the evaluation of individual biomarkers with respect to clinical outcomes as represented by a survival curve (e, left) or global protein expression as represented by a heat map from multiple TMA experiments (e, right).

In 2001, we described the blueprints for developing a means of storing, maintaining and evaluating TMA data using Internet tools and a relational database [6,10]. From this original work has emerged an integrative framework for dealing with all aspects of TMA research (Figure 1). At the center of this process is an internet-based database system referred to as Profiler. This system has the capability of handling/managing a wide variety of sample types from different disease states (i.e., prostate, breast, lung, renal, skin, etc.) and because of its modular design can also store and maintain other types of molecular data including expression array data, karyotype data, and SNP array data. Here within, we describe the process that has developed to deal with TMA experiments and the Profiler system used to manage the data associated with these experiments.

## Results

Over the past 5 years, the Profiler system has developed into a key ingredient into the process for TMA based research. The data included in this process not only includes the alpha-numeric annotations to each histospot but also the images that are associated with each study. The Profiler system has demonstrated a capacity to be extendable to other organ sites besides prostate and modular allowing for analysis of other types of molecular data. To date multiple studies were built/conducted using this system [2,4-6,13-29]. The following is a description of the process that has developed over years of TMA research (Figure 1).

### TMA conceptual research design

TMA design is crucial because it should address the needs of future experiments (Figure 1b, top). Once the TMA design is thought out, two different types of maps are created to assist in the TMA fabrication, a picture map (Figure 1b, lower) and a punching map (not shown). The picture map is a visual representation of the TMA showing a lay out of all the TMA cores. Different tissue types or diagnoses can be shown in colors. The picture map provides orientation. The punching map is needed for taking biopsy cores with the manual arrayer. This map shows the x and y localization of each TMA core and is used during the construction of the TMA.

The physical configuration of the TMA is entered into the Profiler database. All future studies (see below) will maintain this structure and use the information from the punch file to help determine: 1) the type of tissue; 2) targeted diagnosis; 3) the x and y coordinates (i.e., location); and 4) associated research identification code for potential links to clinical and pathology data from external or internal databases.

### Experiments

Each antibody is evaluated using a small test TMA. A test TMA consists of an assortment of different tissues to test the best experimental protocols including pre-treatments and dilutions. The test TMAs are then reviewed by a pathologist and the TMA experiment will then be performed using these optimal conditions (Figure 1c, lower). This process prevents wasting valuable large TMAs.

### Scanning

As described in the methods section, the TMA is scanned and the images are entered into the Profiler database server for evaluation. Initially all slides were scanned with the Bliss system. However, more experiments are being performed using the other imaging systems (described in the methods section).

### Study set up

At an administrative level, the research group determines who should be permitted access to evaluate the TMA. Using Profiler study set up tools, the administrator is given a set of options for the reviewer to have available at time of review. For example, in some studies, staining intensity and percentage intensity are the only parameters that need to be annotated. In another study, nuclear grade may be required. These types of decisions are based on the goals of the study. Once the study is set up, the reviewers are sent a unique user password for them to gain access to studies they are responsible for reviewing. The advantage of this approach is flexibility. As we have seen over the past 5 years, each research group needs to enter data fields that are not readily generalizable to all other groups nor are they relevant. As a specific example, tumor grading systems are often organ specific (e.g., Gleason grading, Fuhrman Grading, etc). Therefore flexibility is critical to allow each group to be able to enter relevant data for their studies. One major disadvantage is that we do not anticipate being able to develop a "standard" set of look up tables to cover every research groups needs and therefore a programmer will be needed to make modifications to the system until it meets an individual groups needs.

### TMA evaluation

Each user gains access to a TMA slide based on their experiments. The reviewer can only see their own folders and not those from other research groups. These safety features are part of the Oracle security tools that are required for e-commerce. As the user views each TMA core image, information about the TMA core is displayed in the region of the screen adjacent to the diagnostic parameters (Figure 1d). The TMA core's X and Y coordinates are displayed with its organ type and target diagnosis. The diagnostic parameters are inputted along with stain intensity, staining location, and path diagnosis using pull down menus. Comments can be entered in a separate free text field. Once a TMA slide evaluation is complete, the user can finalize the evaluation preventing any further changes in the diagnostic parameters. The study design is flexible differing dramatically based on the research group. Clinical and pathology information can be either presented or not. For example, the TMA map is usually available (Figure 1d, lower right). However, one could imagine a study where the investigators may know the design of the TMA and therefore would be biased if this information were available at time of review. We have also found that the tissue diagnosis of each histospot is critical during the evaluation process. When drilling deeper into a block, TMA samples may demonstrate different tissue types (i.e., cancer versus benign) due to tumor heterogeneity.

### Data storage

After each TMA core is reviewed and diagnostic parameters are inputted, the user will submit the information and the next TMA core image will be displayed with all the diagnostic parameters reset for another submission (Figure 1a). The diagnostic data is uploaded onto the data server.

### Analysis

The methods used to analyze TMAs are beyond the scope of this current study. However, it is important to point out that, once a TMA slide has been finalized, all of the raw data is maintained on the Profiler system and available for analysis. The data and images can be queried based on desired features such as all tumor samples staining positive for biomarkers x, y, and z. This feature recalls previous evaluations from the server based on specified parameters (specific cases, tissue cores with particular diagnoses, immunohistochemistry, etc) and displays the data and the corresponding image showing multiple evaluations of one patient adjacent to one another. Query results and single experiment data can be exported as an excel spreadsheet or as a tab delimited text file for further statistical analysis. The system is capable of displaying uploaded data from automated immunostaining results from the Chromavision System, so as all the data stored in the database being related to a specific dataset of interest.

### Additional resources

The Profiler System can be setup to assist in submitted publications as supplementary data showing all information about the study giving raw data as well as the images of the TMA slides. This was recently done with the publication of a biomarker study called Jagged1[30].

### Profiler usage

The Profiler system is currently running at Dana Farber Harvard Comprehensive Cancer and the University of Michigan Comprehensive Cancer Center containing 73.5 K and 95.3 K data points, respectively. 656 TMA slides have been scanned and 63 K histospots evaluated by 68 pathologists. Of the TMAs evaluated, 12 major user groups use the systems including 8 S.P.O.R.E. groups with two groups having directly linked clinical data containing over 500 patients giving immediate access.

## Availability and requirements

One can register for a standard non-anonymous demonstration[31]. This demonstration provides the user with some but not all of the features of the database. The system is accessible via the Internet and currently works on PC computers using Internet Explorer.

## Discussion

In the current study, we describe the evolution of a process that has developed over the past 5 years to use TMAs for biomarker studies. At the center of this process is a relational database tool, Profiler, which allows users to handle all steps of TMA research (Figure 1). The profiler system was originally developed for work in the field of prostate cancer biomarker research but has now been expanded to allow for the analysis of any tissue type. This system has been extensively used by investigators from several academic institutions.

The key feature to Profiler's functionality is the ability to access TMA images over the Internet and enter data in a secure manner. During the course of our first TMA experiments, we observed that there was a high probability of annotating the wrong TMA histospot when multiple pathology reviewers were involved due to density of these TMAs, which typically have 500 TMA cores. We therefore developed a system to present TMA images to the reviewer thus avoiding having to physically identify the TMA histospot by examining the slide. Manually evaluating a TMA slide under the microscope can be a common source of error, since it is reasonably easy to loose track of which samples have been evaluated. Profiler keeps track of which cores have been reviewed so the entire TMAs can be analyzed over a single session or multiple sessions.

The integration of TMA data with other data sets is critical for the development of biomarkers. In version 3.0 of Profiler, other databases can be loaded securely onto the system. Over the past 5 years, several groups with rich clinical and pathology data have used the system to evaluate molecular biomarkers. Profiler's database keeps different datasets separate from each other, maintaining private space for research groups to develop projects. This has become an important issue with some cooperative groups that need to maintain control over their clinical databases based on pre-existing sharing agreements.

Worldwide, one of the guiding principles for any database that maintains clinical data is compliance with local regulatory agencies to protect patient confidentiality. In the United States, HIPPA regulations guide how clinical databases can be integrated with the data stored in Profiler. We have developed two approaches. The first approach is to maintain both datasets separately and then the integration of the Profiler TMA data is performed by the clinical group. They are then typically able to provide a completely integrated dataset without any patient identifiers. This dataset is now ready for analysis. The second approach is to integrate the clinical data into the Profiler system as a separate clinical module. We have done this for several groups and have used Oracle security and administrative tools to ensure that the clinical module is only available for viewing and modifying by the designated clinical groups researchers. All queries require the group leader's approval and each time a query is performed on the clinical data, the time, date, and investigator are recorded and send via email to the group leaders. This monitoring feature is critical to ensure that the clinical database is only used by qualified investigators. The system administrator assigns users different levels of access at different sites using password protected user profiles.

The tracking of standard operating procedures (SOP) is critical for the interpretation of experimental results over time. Therefore the development of a "laboratory book" feature as a new module is critical to the adequate annotation of samples and experiments. We are currently developing the ability to input experiment related data such that a laboratory technician could annotate the staining protocol of each experiment (timing, dilutions, etc). In addition, acquisition settings and image analysis procedures will also be stored in this module of Profiler. Validation studies require specific protocols to reproduce all of the experimental conditions, this module should ensure that this data is available.

Few papers have been published so far on TMA data organization and management. Liu *et al*. [9] presented a system for high-throughput analysis and storage of TMA immuno-staining data, using a combination of commercially available software and novel software. Similarly, Shaknovich et al. [32] proposed a way to manipulate TMA data and images, using commercially available software.

Other academic groups are working on the development of systems integrating commercially available software for the acquisition of digital images and the automatic evaluation of markers with custom solutions for data organization and management.

The Johns Hopkins Tissue Microarray Laboratory has also developed a set of software tools and underlying database structure to manage TMA data[33]. It allows the storage of a wide variety of information related to TMA samples, including patient clinical data, specimens, donor blocks, core, and recipient block information. A dynamic database structure allows users to add custom fields for different organ systems. The client application facilitates automated and manual entry of data related to patients, specimens, tissue blocks, and tissue sub-blocks (individual pathological diagnoses). The system allows users to design their own block arrays. Digital images generated by the Bacus Labs Inc. Slide Scanner (Bacus laboratories, Lombard, IL,) [34] are imported into the database and available for on line visualization and evaluation. Although this system developed separately from the Profiler system, the two groups worked closely initially as part of the S.P.O.R.E. initiative to develop TMAtechnology for translational research[10].

Some other systems have been developed to automatically acquire and evaluate TMA samples, such as the TMALab ™ (Aperio technology) [35], or the Pathfinder™ Morphoscan™ [36]. These solutions work well with high quality TMA slides, usually by superimposing a grid on the panoramic overview of the slide, but they require considerable manual intervention if the TMA samples are not well aligned. These systems are similar to the Bliss and Chromavision systems in that none of these systems identify the histospots automatically. The misalignment of TMA histospots is inevitable due to the way the samples are processed. After a thin 4–5 micron thick section is cut, the histotechnologist floats the sample in a water bath. Even when done by the best-trained technologists, there is slight movement of the samples. Therefore using a grid will never be a practical solution to automate the identification of the samples.

The AQUA system uses an object recognition approach to exactly identify the spatial coordinate of each spot, but lacks in ordering and assigning them to proper patient and/or clinical information based on construction information[7].

The Bioinfomatics Group of ITC in Trento, Italy, has developed an integrated framework for the management of TMA experiment data [37]. This system called TMABoost is a patient centered web based system. It integrates a custom made TMA digital environment for the automatic localization, identification, acquisition and evaluation of TMA single spot. One unique feature of this system is the ability to recognize the histospots taking the TMA map into account [38]. A probability is determined as to the likelihood that a specific histospot is being correctly identified based on the physical map of the TMA. This feature is particularly useful, as during the course of preparing a slide for immunohistochemisty, some histospots may be lost. Ambiguously located histospots would then be excluded or can be manually resolved. This feature using the existing data reduces the chance that TMA histospots are pared with the incorrect x-y coordinate.

Another important feature of TMA based studies is the growing need to share data and information among different institutions (e.g., multi-center studies, clinical trials, etc.). In such a setting, the implementation of standard data exchange protocols becomes critical as up to now there have not been standard approaches to collecting data at different institutions and sometimes even within the same institution and as centralized solutions are not feasible. As a result of several TMA workshops in the area of TMA bioinformatics, TMA Exchange Specifications have been released that allow TMA data to be organized in a self-describing XML document annotated with well-defined common data elements. TMA data exchange specifications have recently been described by Berman et al. [11,39]. Adopting these standards should allow for a seamless integration of public TMA databases. The public sharing of TMA data following publication of data, similar to standards developed for expression array data exchange should facilitate biomedical research. As part of a National Biospecimens Pilot Project[40], the 11 Prostate Cancer S.P.O.R.E. groups will adopt these standard and work with the Cancer Biomedical Informatics Grid (caBIG) program [41] to ensure that these standards remain compliant. Future versions of this system will adopt an ontology system developed in collaboration with the caBIG program. Currently our vocabulary and definitions have been more locally defined and will need to be adjusted as these standards become better defined.

Profiler can be set up at academic institutions that would like to use this system. Although the system is currently written for Oracle, the Profiler application can be deployed to Open Source Software. Apache Web Server [42] and Tomcat Engine [43] are currently Open Source Software. All front end codes for Profiler application can modify to support to Open Source. There are several Open Source databases such as MySQL [44] or PostgreSQL [45] that can be integrated with Profiler application with small modification of JDBC drivers and Java codes. We are currently looking for an Open Source Image application to support TMA images. The Profiler system is available to all academic intuitions using a standard academic licensing agreement. This procedure is intended to make the system widely available and is required by our institutions based on their established intellectual properties policies. Any interested investigators should contact the corresponding author to begin the licensing procedure. Groups using a different database system (e.g., MySQL) may need to modify the database scripts and perform additional testing. But in general both the code and the relational database schema are highly portable.

## Conclusion

In summary, we have described a process to deal with TMA experiments using a system called Profiler. The TMA module of Profiler allows for a secure and reliable way to evaluate and store TMA images over the Internet. The database allows for integration of other types of dataset including clinical and pathology data. The system has been extensively used for the evaluation of biomarkers and has proven to be reliable and stable at two Comprehensive Cancer Centers. This system is freely available to academic intuitions through a standard licensing agreement.

## Methods

### TMA construction

TMA technology allows for the placement of 400–800 0.6 mm diameter tissue samples into a single standard tissue block[1]. High-density TMA are assembled using either the manual or automated tissue puncher/array. These instruments consist of thin-walled stainless steel needles with an inner diameter of approximately 600 μm and stylet used to transfer and empty the needle contents. The assembly is held in an X-Y position guide that is manually adjusted by digital micrometers. Small biopsies are retrieved from selected regions of donor tissue and are precisely arrayed in a new paraffin block (Figure 1c). The study pathologist selects the area of interest used for placement into the TMAs by circling the area on a standard H&E glass slide representative of the tissue block.

All experiments that can be performed on standard tissue sections can also be performed using TMAs (Figure 1c). Therefore DNA, RNA or protein expression can be evaluated on TMAs by using FISH, *in situ *hybridization, immunohistochemistry or immunoflourescene.

### In situ protein expression: human versus automated evaluation

The evaluation of tissue samples have been traditionally performed by pathologists using microscopy. Usually staining intensity is scored with a 4 nominal point scale (negative, weak positive, moderate positive, strong positive) and the stained area is scored as a percentage value. However, the human eye, even if trained, cannot detect subtle differences in staining intensity on a continuous scale, in particular at very low and very high levels of the scale. Moreover the nominal categories are highly subjective and inter- intra-observer agreement is poor. In contrast, quantitative detection of immunostaining provides continuous measures ensuring reproducibility. The continuous quantitative measurements obtained by semi-automatic evaluation can better detect the information of the investigated target, allowing the detection of subsets of tumors not seen using human/pathologist based assessments as demonstrated by Camp et al. using immunoflourescene[7].

Currently, there are no fully automated methods of evaluating protein expression by immunohistochemisity without the concurrent evaluation by a pathologist. Depending on how carefully the area of interest is circled the typical 0.6 mm diameter core may or may not contain this sample. Thus the targeted (pre-transfer diagnosis) sample is often but not always what ends up on the TMA recipient block. Further complicating this is the possibility that as one cuts deeper into the TMA block, the lesion of interest due to tissue heterogeneity may not be consistent throughout. Therefore, it is critical along every step of the evaluation process that a pathologist or someone trained at interpreting histologic samples confirms the diagnosis of the sample.

### Profiler system (version 3.0)

Profiler is a Java-based web application designed to provide flexible access to TMA data consisting of scanned images and the associated evaluations. Profiler was initially developed through NCI funding as part of a supplement to the Prostate Cancer S.P.O.R.E. at the University of Michigan in 2001. Version 3.0 has been set up at the University of Michigan Comprehensive Cancer Center and at the Dana Farber Harvard Cancer Center.

A fundamental principle for the Profiler system is compliance with current regulatory guidelines to ensure patient data security and confidentiality. In addition to general guidelines such as those established by Health Insurance Portability and Accountability Act (HIPAA), standards for sharing and transmitting data have been implemented by such groups as the Cooperative Prostate Cancer Tissue Resource (CPCTR)[46] to ensure compliance with the Cancer Biomedical Informatics Grid[41], an initiative of the NCI.

Profiler is implemented as a Java application running on top of the Apache web server and of the Tomcat Java engine. This architecture, based on open standards, is reliable, well-tested and highly scalable, and allows the users to connect to the system using any Java-compliant web browser rather than requiring ad-hoc client programs. It also allows the developers to take advantage of the security features of modern Web tools, including SSL secure sockets which provide strong encryption of the data passing between the client and the server.

The heart of the Profiler system is the Java middleware that implements the "business logic" of the application and that manages the entire user session, using Java Beans to track the various parameters of the session. Java Server Pages (JSP) functions are used to generate HTML pages that are specific for the user, on the basis of his/her access permissions, and of the tool he/she is using within the application. The customized user interface presents the user with only the menus and tools he/she is allowed to see for the intended task, thus reducing confusion and preventing entry errors.

The Oracle relational database represents the foundation of the system, offering high performance and a high level of security. Oracle also supports the full suite of JDBC, SQL and XML tools to link to data in other databases. Support of XML facilitates the interchange of data with collaborators and allows us to provide links, where appropriate, to other source-of-truth databases (the clinical LIS, for example), rather than duplicate the data in our databases. Data that is stored locally includes TMA histospot information, pathology evaluation results, and TMA image information.

Image handling is fully integrated within the TMA Profiler application. TMA images are stored in JPEG format and are displayed in the interface page by a specialized Java image viewer that allows the user to zoom into the image or to move it in real time. The viewer is optimized to efficiently download images from the image server, and to work at different resolution levels according to the amount of data received.

An example of how the TMA Profiler system is currently being used can be seen at in a website recently set up for public viewing of the expression of JAGGED1 in prostate cancer[30].

The Profiler System runs on two dual-processor servers, each with one Gigabyte of RAM memory. The first server, running the Microsoft Windows 2000 operating system, hosts the imaging server based on the Webslide Server package (Bacus Labs, Lombard, IL). The second server, running the Red Hat ^® ^Linux operating system, hosts the web server (Apache), Jakarta Tomcat, the Oracle database and the JSP code for the entire application.

### TMA data structure

The Profiler system consists of modules for the organization and analysis of molecular data. The TMA module was developed from a previously described Microsoft Access relational database[10]. The current version 3.0 of Profiler utilizes a centralized relational database performing automatic queries, which can be accessed over the Internet. Profiler utilizes external modular data structures as a means to link TMA pathology or clinical data. When new data such as kayotype data is added into the database, it is stored in the form of a module. These modules give Profiler the ability to incorporate relational information without having to restructure the system. Each module can be linked together using a unique linkage key during the time the data is accessed. Figure 1a shows a schematic representation of how each module is used in accordance with the relational database. To incorporate a new module, a linkage key must be included in the module to serve as a means to relate all the appropriate information. Data queries can only be executed on a permission basis. Different datasets, while related, are kept separate from non-collaborating researchers. The Profiler system is adaptable to the changing research demands on a project basis.

In the initial iteration of this database, only prostate organ specific data was tracked[10]. As the system expanded to other users studying other organ systems, we developed look up tables specific to these organ sites such as lung, breast, pancreatic, head and neck, and the hematopoietic system. A specific list of possible diagnoses and grading parameters are kept separate for each organ type into look-up tables. The organ specific or project specific parameters can be added dynamically so that the system does not have to be adjusted to accommodate the changes. Each tissue specific list can be updated while only displaying chosen fields during the evaluation. During the evaluation stage, the user interface alters its diagnostic parameters based on the organ tissue of the TMA core. As an example, the diagnostic parameters would change based on each TMA core of a multi-tumor array.

### Image acquisition

Digital images are acquired for each histospot of each TMA experiment. The reason is twofold. On one hand, TMA digital images complete the information stored for each experiment. On the other hand, the availability of good quality digital images allows for user-friendly TMA sample evaluations on a screen.

During the initial development of the Profiler system, we selected the BLISS Imaging system of the Bacus Laboratory Inc. Slide Scanner (BLISS^®^) (Bacus Laboratory Inc., Lombard, IL). It is a semi-automated system to acquire high resolution images, as previously described [10]. The images are captured in JPEG format and are uploaded onto the Profiler server via FTP (file transfer protocol). Once the images are uploaded, the server is setup to display the images according to the TMA specifications. Each TMA file is constructed using a data file containing information about each tissue sample in the array.

### External imaging analysis systems

Profiler V3.0 has the capability to integrate data from external image analysis systems into its database. For example, we set up an integration module for the Automated Cellular Imaging System (ACIS II) from Clarient Chromavision Medical Systems (San Juan Capitstrano, CA). This system works on quantitative evaluation of protein expression on bright field. It creates a panoramic overview of the sample, acquires single spot images and then performs quantitative evaluation. Images can be exported from the Chromavision system as JPG, TIFF, or GIF files. We are also developing import filters for data generated from the Automated Quantitative Analysis (AQUA) system (HistoRX Inc., New Haven, CT). AQUA is a fluorescent based imaging system, which is capable of quantitating sub-cellular localization of protein expression [7,26].

### Web-based TMA evaluation interface

After a TMA experiment has been completed (Figure 1c) and the TMA slide is scanned using one of the imaging systems described above, the pathologist reviewer enters a folder with a collection of all images associated with the given study. Once the review process begins, the reviewer can either select images to review or will more routinely go from the first image and be presented all of the captured images for that study (Figure 1d). Each TMA core image is viewed one at a time. The reviewer can annotate the tissue, assigning the appropriate diagnosis, staining intensity, and other organ specific parameters. The presentation of which parameters are required will vary from study to study and therefore are entirely flexible. A typical interface for the TMA evaluation process is presented in Figure 1d. Each high resolution TMA core image is displayed with the option of zooming in and out and panning over the image, simulating the functionality of looking at a slide under the microscope. A corresponding hematoxylin and eosin stained (H&E) image can be viewed to assist in the evaluation. Diagnostic parameters for each sample are stored and the next spot is displayed. A small legend map of the array can be displayed showing the status of each core whether it has been evaluated or flagged for review (Figure 1d, lower right). Evaluations can be completed over multiple sessions with the legend map indicating which cores have yet to be reviewed. The legend map is used to navigate to any particular core in the array. Completed evaluations are time stamped and associated with a particular evaluator. A demonstration of this interface is available for evaluation[31].

### Data privacy and security

Once a TMA slide is scanned, TMA core images can be viewed online using Profiler's evaluation interface. To log in, each user must enter their username and password at Profiler's home page in order to access their slides. Usernames and passwords are created only after a user has registered an account. TMAs displayed on Profiler can be evaluated from any computer with an Internet connection.

In the United States, the integration of Clinical and Pathology data from external databases must be compliant with current HIPAA of 1996 (Public law 104–191)[47]. In brief, we use a research code provided by the designated curators of each of the clinical databases. Each clinical and pathology database requires its own Institutional Review Board (IRB) Protocol number prior to incorporation into Profiler. This is in agreement with the IRB policies and procedures at both of the sponsoring institutions. According to HIPAA, Sec. 164.514(a)-(c), the Privacy Rule permits a covered entity to de-identify protected health information (PHI). The unique, de-identified patient ID therefore can not use any 18 PHI data elements specified by the Privacy Rule. The resulting de-identified patient ID is then the only master ID entered into the central Profiler database in conjunction with associated clinical, pathologic, genomic, and other sample related data. The de-identified patient ID is also used for identifying biological samples during all aspects of experimentation, data collection, and data viewing by researchers. Re-linkage of de-identified consortium patient ID to actual patient identifiers is manually possible only by specific representative of each research group with manual control of the local linkage list and under guidance of their home IRB policies.

## Authors' contributions

RK developed the imaging protocols for Profiler.

FD was involved in the design process of Profiler modules

JT and AR expanded the Profiler system from its original form and made it extendable for broader (i.e. generic) use.

RS, DFG, VM, AMC, MAR were involved in the original development of the Profiler system.
